# Role of 18F-Fluorodeoxyglucose–Positron Emission Tomography/Computed Tomography Imaging in the Prediction of Prognosis in Patients With Indolent Lymphoma: Prospective Study

**DOI:** 10.2196/24936

**Published:** 2021-11-12

**Authors:** Nawal Faiez AlShehry, Raja Shanker, Syed Ziauddin Ahmed Zaidi, Fahad AlGhmlas, Ibraheem Hussein Motabi, Shahid Iqbal, Ahmad Ali Butt, Hassan AlShehri, Imran Khan Tailor, Syed Yasir Altaf, Mubarak AlGhamdi, Mohammed Marie, Mansour AlFayez, Kamal Al Zahrani, Mohammed Dwaimah, Tahani Al-Halouli, Wafaa Al-Shakweer, Maied Zaher AlShehery, Abdul Rehman Zia Zaidi, Atta Munawar Gill, Belal Mohammed Albtoosh, Musab Ahmed

**Affiliations:** 1 Department of Adult Hematology/Bone Marrow Transplantation King Fahad Medical City Riyadh Saudi Arabia; 2 Department of Radiology and Nuclear Medicine King Fahad Medical City Riyadh Saudi Arabia; 3 Department of Pathology and Laboratory Medicine King Fahad Medical City Riyadh Saudi Arabia; 4 Department of Palliative Care King Fahad Medical City Riyadh Saudi Arabia

**Keywords:** positron emission tomography, lymphoma, prognosis, indolent lymphoma, SUVmax, Deauville criteria

## Abstract

**Background:**

The role of fluorodeoxyglucose–positron emission tomography/computed tomography (FDG-PET/CT) in indolent lymphoma has been minimally studied.

**Objective:**

This study aims to assess the value of FDG-PET/CT in predicting the prognosis of indolent lymphoma.

**Methods:**

We prospectively recruited 42 patients with indolent lymphoma. A total of 2 patients were excluded, and 40 underwent baseline PET/CT and follow-up at various time points. A total of 9 patients were observed only, 7 received 4 doses of rituximab alone, and 24 received chemoimmunotherapy. Metabolic response on follow-up PET/CT was assessed using the maximum standardized uptake value (SUVmax) and Deauville criteria (DC). We aimed to obtain the best SUVmax and DC to predict optimal survival rates, risk stratification, and optimize therapeutic strategies. The mean follow-up from the initial diagnosis was 33.83 months.

**Results:**

SUVmax <4.35 at interim PET/CT provided the best discrimination, with a progression-free survival (PFS) of 100% and a median survival time of 106.67 months compared with SUVmax ≥4.35 (*P*=.04), which had a PFS of 43.8% and a median survival time of 50.17 months. This cutoff was also valuable in predicting overall survival at baseline, that is, 100% overall survival with baseline SUVmax <4.35, versus 58.4% for SUVmax ≥4.35 (*P*=.13). The overall survival of patients with a baseline DC score <3.0 was 100%, with a median overall survival of 106.67 months.

**Conclusions:**

We demonstrated the utility of PET/CT in indolent lymphomas. SUVmax (<4.35 vs ≥4.35) on interim PET/CT performed best in predicting PFS.

## Introduction

### Background

Indolent lymphomas are a heterogeneous group of mature B-cell non-Hodgkin lymphomas, characterized by a slow growth rate and a tendency to relapse. The frequencies of different types of indolent lymphomas based on the World Health Organization classification 2008 are follicular lymphoma (FL) (29%), small lymphocytic lymphoma (SLL) and chronic lymphocytic leukemia (CLL) (12%), mucosal-associated lymphoid tissue lymphoma (9%), nodal marginal zone lymphoma (2%), splenic marginal zone lymphoma (0.9%), and lymphoplasmacytic lymphoma (1.4%) [1]. The prognosis of indolent lymphoma varies according to the stage, clinical features, histology, immunophenotyping, response to first-line therapy, and the duration of remission [1]. Prognostic scores have been proposed specifically for the common types of FL [2,3], CLL [4], lymphoplasmacytic lymphoma [5], and splenic marginal zone lymphomas [6].

Indolent lymphomas mostly relapse to the same type of lymphoma or undergo histological transformation to more aggressive types of lymphomas. ^18^F-Fluorodeoxyglucose (FDG)–positron emission tomography (PET) has been valuable in evaluating the response to therapy, in addition to the diagnosis, staging, and surveillance of lymphoma patients. The maximum standardized uptake value (SUVmax) is a semiquantitative index used to objectively interpret the metabolic activity of various tissues (eg, neoplastic tissues) on PET/computed tomography (CT) scans. An international consensus helped establish a simpler, robust, and reproducible criterion for PET/CT interpretation in Hodgkin lymphoma (HL). The resultant 5-point Deauville criteria (DC) score helped in risk stratification, and strategizing adaptive therapies in HL, based on interim PET/CT findings with very high sensitivity and specificity. The DC established the role of PET/CT at the initial staging as well as at the end of the treatment [7,8]. Notably, the role of FDG-PET/CT in management has been scarcely studied internationally and is limited to FL and mantle cell lymphoma (MCL) [9,10].

The treatment of indolent lymphoma depends on whether the disease is localized or in an advanced stage. Therapeutic modalities range from watchful waiting to monoclonal anti-CD20 antibody therapy, conjugated-radiolabeled monoclonal antibody therapy, or more intensive chemoimmunotherapy and radiation therapy as well as high-dose chemotherapy and stem cell transplantation [11].

Superior tools for prognostication would help in risk stratification at the initial diagnosis or at the mid or end of treatment. High-risk patients, who are likely to relapse or undergo transformation to aggressive lymphomas, would be qualified to receive more intense therapeutic approaches and closer follow-up, particularly in the era of newly emerging targeted therapy for lymphomas. Emerging data suggest that metabolic response to FDG-PET/CT is superior in comparison with other tools currently available, especially in early prognostication of HL and diffuse large B-cell lymphoma (DLBCL) [12-14]; the same may hold true in indolent lymphoma. In particular, the semiquantitative SUVmax has been found to objectively supplement visual interpretation of FDG-PET/CT [15], during and after therapy for HL and aggressive non-HL, and interim FDG-PET/CT was found to be an independent, strong predictor of progression-free survival (PFS) in HL [16]. Furthermore, in DLBCL, SUVmax has a high prognostic value and is strongly correlated with survival [12]. Similarly, SUVmax in DLBCL significantly improved prognostication after the 1st line of chemotherapy [12].

Although most indolent lymphomas show low metabolic activity of glucose, and hence low SUVmax, FDG-PET/CT has been found to be helpful in staging and evaluating histological transformation. Moreover, in FL, FDG avidity appears to be generally more pronounced than in other types of indolent lymphomas. PET/CT is also more accurate than conventional imaging for initial staging, often prompting significant management changes [17].

The results of a clinical trial [17] found that FDG-PET/CT should be considered as a new standard for response assessment of FL and guiding response-adapted therapy. In another study, end-of-treatment PET/CT findings were found to be predictive of overall survival in patients with FL [18]. A review by El-Galaly et al [19] highlighted the role of PET/CT in staging and its impact on modern treatment selection for lymphomas, including the indolent group of lymphomas.

### Objective

We prospectively studied the utility of FDG-PET/CT in the management of indolent lymphomas at our tertiary care institution in Saudi Arabia. This study aims to assess the value of FDG-PET/CT in predicting the prognosis of indolent lymphomas.

## Methods

### Study Design

This was a prospective study conducted at King Fahad Medical City, Riyadh, Saudi Arabia, after approval by the Institutional Review Board under study number 15-262.

### Patient Population and Sampling Technique

All newly diagnosed adult patients with different types of indolent lymphomas were enrolled in the study after obtaining informed consent from August 2014 to May 2018. A 1-year follow-up scan for the last recruited patient was completed in May 2019. Data analysis was completed in February 2020.

### Inclusion Criteria

All patients aged ≥14 years (as patients in this this age group are treated by the adult team in our hospital), a confirmed diagnosis of indolent lymphoma (other than FL grade 3b), any stage (Ann Arbor I, II, III, IV and in CLL, Rai stage 0-IV), and those who underwent a PET/CT scan before treatment or start of observation were included in the study.

### Exclusion Criteria

Patients with grade 3b FL were excluded. Patients could withdraw from the study for any reason.

### Treatment Guidelines

On the basis of the initial histology and stage, the treatment options were as follows:

- Chemotherapy+Immunotherapy (chemoimmunotherapy)
- Rituximab only
- Observation only

All these factors were considered for finding any significant correlations between the PET/CT scan and the specified endpoints.

### Primary Objective

To assess the utility of FDG-PET/CT in prognostication (based on SUVmax and DC) at various time points (baseline, midtreatment, end of treatment, and 1-year follow-up).

### Secondary Objectives

- To evaluate the role of FDG-PET/CT in adaptive therapies, that is, intensive chemotherapy or targeted therapy.
- To evaluate the utility of PET/CT in situations such as disease progression or transformation of indolent lymphoma to DLBCL.

### Data Collection Procedures

The patients were managed according to current standards of practice. Case report forms were completed for all patients with data entry at various phases of the study. The clinical data were collected from electronic medical records.

All newly diagnosed patients (proven by histopathology) had pretreatment baseline evaluation with FDG-PET/CT and follow-up PET/CT, that is, interim PET/CT, end-of-treatment PET/CT, and PET/CT 1 year later. FDG uptake was measured by SUVmax normalization for total body weight, in addition to the DC. The DC is a five-point scale that uses the mediastinum and liver as reference organs. The patients were followed prospectively to assess the prognostic value of early and end-of-treatment PET/CT in these patients.

The primary endpoints of this study were the determination of PFS in correlation with PET/CT findings (SUVmax and DC) at different time points.

### FDG-PET/CT Procedure

All PET/CT scans were acquired per accepted protocol following a tracer dose of 8-12 mCi of FDG for adults, or weight-adjusted dosage in smaller patients on the same GE 960 STE scanner (General Electric, Waukesha, WI, USA). All scans were performed using the same image acquisition and reconstruction protocol. PET/CT was performed from the vertex of the skull to the knees 60 minutes after injection and took an average time of 30 min. Both the uncorrected and attenuation-corrected images were reviewed. All PET/CT scans were reviewed visually and semiquantitatively (using the SUVmax and DC) and interpreted blindly by 2 experienced nuclear medicine physicians. The PET/CT images and interpretations were reviewed in clinical context by experienced hematologists.

### Statistical Analysis

All the data were analyzed using SPSS, version 25 (IBM Corporation). The primary endpoints and outcomes, with complete follow-up assessment of the study, were evaluated by the difference in response rates between patients treated with chemoimmunotherapy and rituximab alone. The log-rank test was applied to determine the time survival between 2 or more independent groups. Kaplan–Meier (KM) survival curves were used to estimate the PFS and overall survival of the patients. Relapse-free survival (RFS) was also estimated for patient groups treated with rituximab and chemoimmunotherapy. *P*<.05 was considered as statistically significant.

## Results

### Overview

**Figure 1:**
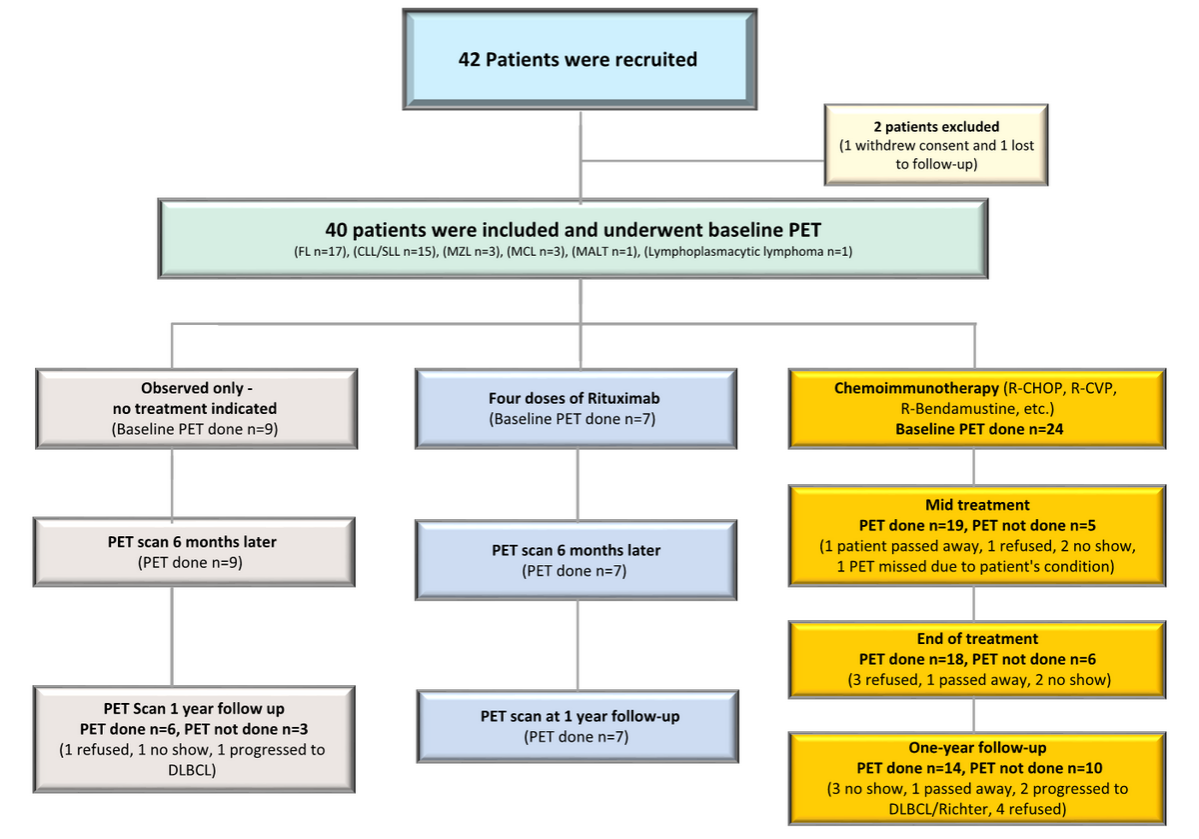
Patient allocation and schema of follow-up during the study. CLL: chronic lymphocytic leukemia; DLBCL: diffuse large B-cell lymphoma; FL: follicular lymphoma; MALT: mucosa-associated lymphoid tissue lymphoma; MCL: mantle cell lymphoma; MZL: marginal zone lymphoma; PET: positron emission tomography; SLL: small lymphocytic lymphoma; R-CHOP: rituximab–cyclophosphamide, doxorubicin hydrochloride, Oncovin, prednisone; R-CVP: rituximab–cyclophosphamide, vincristine sulfate, prednisone.

Of the 42 patients initially enrolled in the study, 40 were analyzed. Two patients were excluded: one due to withdrawal of consent and the other was lost to follow-up (Figure 1).

### Patient Characteristics

The study population included 68% (27/40) men and 33% (13/40) women. Patients’ ages ranged from 28 to 88 years with a mean of 59.10 (SD 14.78). The diagnoses included FL (17/40, 43%), SLL and CLL (15/40, 38%), marginal zone lymphoma (3/40, 8%), MCL (3/40, 8%), mucosal-associated lymphoid tissue lymphoma (1/40, 3%), and lymphoplasmacytic lymphoma (1/40, 3%; Table 1).

**Table 1:** Basic demography and clinical characteristics of the patients (N=40).

Characteristics			Participants
Age at 1st diagnosis (years)^a^, mean (SD)			59.10 (14.78)
**Gender, n (%)**			
	Male	27 (68)	
	Female	13 (33)	
**Therapeutic strategy, n (%)**			
	Chemoimmunotherapy	24 (60)	
	Rituximab	7 (18)	
	Observation	9 (23)	
**Diagnosis, n (%)**			
	Follicular	17 (43)	
	SLL^b^ and CLL^c^	15 (38)	
	Marginal zone lymphoma	3 (8)	
	Mantle cell	3 (8)	
	MALT^d^	1 (3)	
	Lymphoplasmacytic lymphoma	1 (3)	
**Lymphadenopathy,** **n (%)**			
	Yes	31 (78)	
	No	9 (23)	
**Organomegaly, n (%)**			
	Yes	14 (35)	
	No	26 (65)	
**Bone marrow infiltration at diagnosis, n (%)**			
	Yes	28 (70)	
	No	9 (23)	
	Not done	3 (8)	
**Bulky disease, n (%)**			
	Yes	5 (13)	
	No	35 (88)	
	Not done	0 (0)	
**Final staging, n (%)**			
	Early stage	11 (28)	
	Advanced-stage disease	29 (73)	
**IPI^e^, n (%)**			
	Not applicable	38 (95)	
	Intermediate	1 (35)	
	High	1 (3)	
**FLIPI^f^, n (%)**			
	Not applicable	24 (60)	
	Low	1 (35)	
	Intermediate	3 (8)	
	High	12 (30)	
**Rituximab with 1st line, n (%)**			
	Yes	31 (78)	
	No	3 (8)	
	Not applicable	6 (15)	
**Rituximab maintenance, n (%)**			
	Yes	9 (23)	
	No	22 (55)	
	Not applicable	9 (23)	
**Relapse status, n (%)**			
	Yes	5 (13)	
	No	29 (73)	
**Outcome, n (%)**			
	Died	5 (13)	
	Alive	34 (85)	

^a^The median age of participants is 61 years (range 28-82 years).

^b^SLL: small lymphocytic lymphoma.

^c^CLL: chronic lymphocytic leukemia.

^d^MALT: mucosal-associated lymphoid tissue.

^e^IPI: international prognostic index.

^f^FLIPI: follicular lymphoma international prognostic index.

All 40 patients underwent baseline PET/CT. On the basis of clinical presentation, the patients were managed per accepted protocol as follows: either observed only (n=9), received 4 doses of rituximab alone (n=7), or received chemoimmunotherapy (R-CHOP [rituximab-cychlophosphamide, hydroxydaunorubicin, vincristine and prednisone], R-CVP [cituximab-cychlophosphamide, vincristine and prednisone], and R-bendamustine; n=24). Most of the patients underwent scheduled FDG-PET/CT scans at midtreatment (only chemoimmunotherapy arm), at 6 months, and at 1 year. However, due to logistics, we could not obtain PET/CT scans in a few patients in the later phases on 16 occasions (Figure 1). For the overall study population, SUVmax and DC scores at baseline, interim, and end of treatment per 1 year observation as well as 1 year after chemotherapy are summarized in Table 2.

**Table 2:** Descriptive analysis of the study population for maximum standardized uptake value (SUVmax) and Deauville criteria.

Characteristics	Value, median (range)	Value, mean (SD)
SUVmax baseline	6 (2-24)	8 (5)
SUVmax interim	4 (1-25)	5 (4)
SUVmax Eed of treatment per 1 year observation	3 (1-25)	5 (5)
SUVmax at 1 year after last C or chemotherapy	4 (2-14)	5 (3)
Deauville baseline	5 (2-5)	4 (1)
Deauville interim	3 (1-5)	3 (1)
Deauville end treatment per 1 year	2 (1-5)	2 (1)
Deauville at 1 year after last chemoimmunotherapy	2 (1-5)	3 (1)

The mean time for follow-up of chemoimmunotherapy patients was 33.24 months (SD 18.2); whereas it was 35.99 months (SD 11.65) and 38.83 months (SD 30.82) for rituximab and observation groups, respectively.

Due to the limited sample sizes of different subsets of indolent lymphomas, we chose to compare patients with FL (n=17) against a subset of patients with nonfollicular indolent lymphoma (n=23) as presented in Table 3. Bulky disease was seen in 29% (5/17) patients with FL, whereas 71% (12/17) of patients with FL had high follicular lymphoma international prognostic index (FLIPI) scores. Of the 40 patients, 73% (29/40) had advanced-stage disease, more so in the FL group with 88% (15/17) patients than in the nonfollicular indolent lymphoma group, with 61% (14/23; *P*=.06) patients. Lymphadenopathy was observed in 94% (16/17) patients with FL, compared with 65% (15/23) in the nonfollicular indolent lymphoma patients (*P*=.03). However, organomegaly was more common in the non-FL group (*P*=.05) as presented in Table 3.

**Table 3:** Relationship between the diagnosis of follicular lymphoma (FL) versus non-FL type of indolent lymphomas and characteristics of patients (N=40).

Characteristics			Follicular (n=17), n (%)		Nonfollicular (n=23), n (%)		*P* value	
**Gender**								.27
	Male	12 (71)		15 (65)				
	Female	5 (29)		8 (35)				
**B symptoms**								.68
	Yes	7 (41)		8 (35)				
	No	10 (59)		15 (65)				
**Lymphadenopathy**								.03
	Yes	16 (94)		15 (65)				
	No	1 (6)		8 (35)				
**Organomegaly**								.048
	Yes	3 (18)		11 (48)				
	No	14 (82)		12 (52)				
**Bone marrow infiltration at diagnosis**								.24
	Yes	12 (71)		16 (70)				
	No	5 (29)		4 (17)				
	Not done	0 (0)		3 (13)				
**Bulky disease**								.005
	Yes	5 (29)		0 (0)				
	No	12 (71)		23 (100)				
**Final staging**								.06
	Early stage	2 (12)		9 (39)				
	Advanced-stage disease	15 (88)		14 (61)				
**IPI^a^**								.50
	Not applicable	17 (100)		21 (91)				
	Intermediate	0 (0)		1 (4)				
	High	0 (0)		1 (4)				
**FLIPI^b^**								.001
	Not applicable	1 (6)		23 (100)				
	Low	1 (6)		0 (0)				
	Intermediate	3 (18)		0 (0)				
	High	12 (71)		0 (0)				

^a^IPI: international prognostic index.

^b^FLIPI: follicular lymphoma international prognostic index.

**Figure 2:**
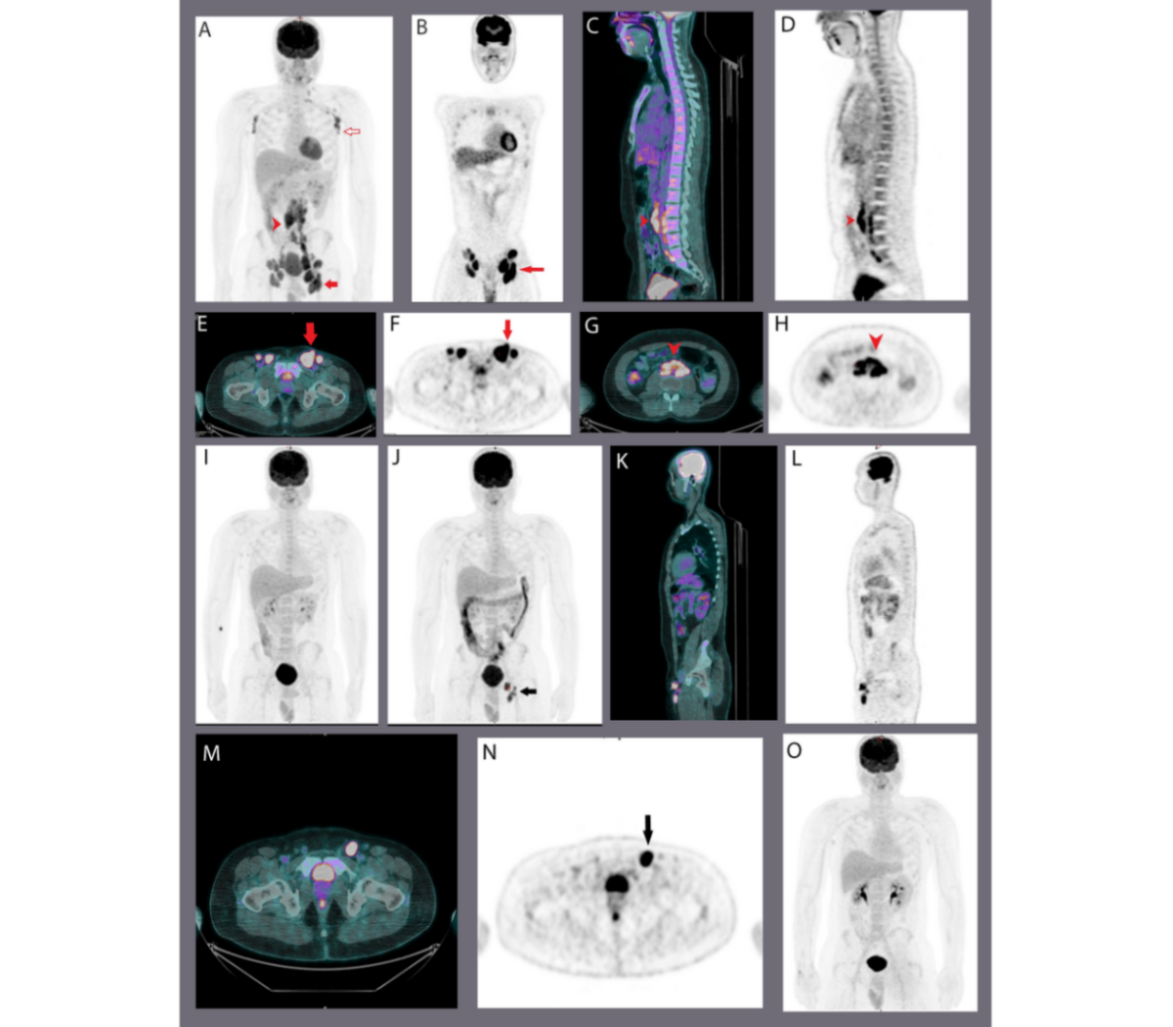
Sequential fluorodeoxyglucose–positron emission tomography/computed tomography scan findings in subject number 2, a case of follicular lymphoma.

As expected, the advanced stage of indolent lymphoma had a higher SUVmax in comparison with early-stage indolent lymphomas (Table 4). Patients with a DC score>3.0, at baseline, had higher lactate dehydrogenase compared with those with a DC score ≤3.0 (20/23, 87% vs 3/23, 13%; Table 5). Patients with FL had higher SUVmax and DC scores than all other nonfollicular indolent lymphomas (Table 6). Twelve patients with FL had bone marrow (BM) involvement on biopsy, but in one of these patients, BM involvement could not be detected on PET/CT (Figure 2).

Patients with a Deauville score >3.0 at baseline had high lactate dehydrogenase (20/23, 87%) compared with those with a Deauville score ≤3.0 (3/23, 13%).

**Table 4:** Distribution of patients according to the maximum standardized uptake value (SUVmax), Deauville and final staging (N=40).

Characteristics			Disease staging, n (%)					*P* value	
			Early stage		Advanced-stage disease				
**Group SUVmax baseline**									<.001
	<4.35	9 (82)		4 (14)					
	≥4.35	2 (18)		25 (86)					
**Group SUVmax baseline**									.007
	<9.41	11(100)		16 (55)					
	≥9.41	0 (0)		13 (45)					
**Group Deauville baseline**									<.001
	≤3.0	8 (73)		2 (7)					
	>3.0	3 (27)		27 (93)					
**Group Deauville baseline**									<.001
	≤4.0	10 (91)		7 (24)					
	>4.0	1 (9)		22 (76)					

**Table 5:** Distribution of patients according to the maximum standardized uptake value (SUVmax), Deauville score, and lactate dehydrogenase (N=40).

Characteristics		LDH^a^, n (%)			*P* value
		≤220 (normal; n=17)	>220 (high; n=23)		
**SUVmax baseline**					.31
	<4.35	7 (41)	6 (26)		
	≥4.35	10 (59)	17 (74)		
**SUVmax baseline**					.29
	<9.41	13 (76)	14 (61)		
	≥9.41	4 (24)	9 (39)		
**Deauville baseline**					.04
	≤3.0	7 (41)	3 (13)		
	>3.0	10 (59)	20 (87)		
**Deauville baseline**					.25
	≤4.0	9 (53)	8 (35)		
	>4.0	8 (47)	15 (65)		

^a^LDH: lactate dehydrogenase.

**Table 6:** Distribution of patients according to the maximum standardized uptake value (SUVmax), Deauville, and diagnosis (N=40).

Characteristics		Diagnosis, n (%)			*P* value
		Follicular	Nonfollicular		
**Group SUVmax baseline**					.002
	<4.35	1 (6)	12 (52)		
	≥4.35	16 (94)	11 (48)		
**Group SUVmax baseline**					<.001
	<9.41	6 (35)	21 (91)		
	≥9.41	11 (65)	2 (9)		
**Group Deauville baseline**					.02
	≤3.0	1 (6)	9 (39)		
	>3.0	16 (94)	14 (61)		
**Group Deauville baseline**					.006
	≤4.0	3 (18)	14 (61)		
	>4.0	14 (82)	9 (39)		

Figure 2 shows the images of the patient from initial presentation (images A-H) until complete metabolic remission. Maximum intensity projection (MIP; image A) and multiplanar PET/CT demonstrated extensive hypermetabolic lymphadenopathy above and below the diaphragm. The arrowhead indicates retroperitoneal lymphadenopathy, and the solid arrow indicates left inguinal lymphadenopathy (A, MIP; B, coronal PET; C, sagittal fused PET/CT; D, sagittal PET; E, axial fused PET/CT; F, axial PET; G, axial fused PET/CT; and H, axial PET). BM involvement was not observed. The patient achieved complete metabolic remission of previously noted residual hypermetabolic lymphadenopathy above and below the diaphragm after 6 cycles of R-CVP and rituximab maintenance (image I, MIP). He relapsed within 21 months with interval development of FDG-avid left inguinal lymphadenopathy (images J-N). Solid arrow: left inguinal lymphadenopathy (J, MIP; K, sagittal fused PET/CT; L, sagittal PET; M, axial fused PET/CT; and N, axial PET). After involved-field radiation to the inguinal area, the patient achieved complete metabolic remission of previously noted left inguinal lymphadenopathy (image O, MIP).

**Figure 3:**
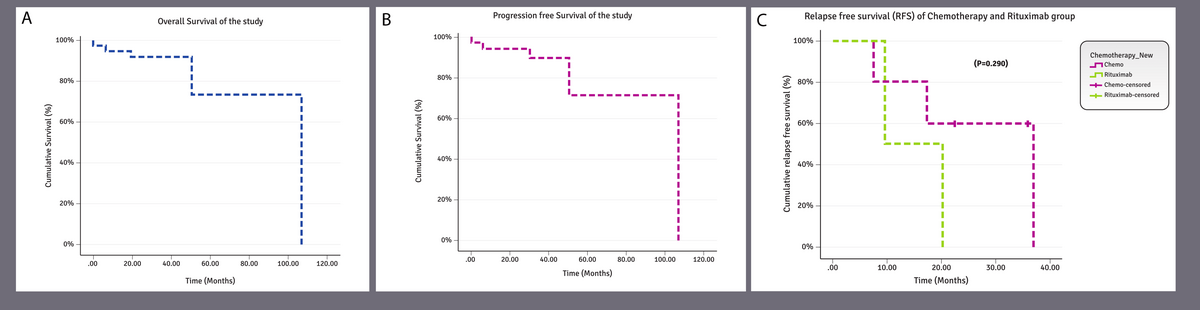
(A) Overall survival of all the study patients (N=40). The cumulative overall survival rate of the study was 73.4%. However, it was not possible to obtain the median survival time of this curve, as the censorship was less than 50% but the estimated overall mean survival time was recorded as 88.29 (range 66.28–110.31) months. (B) Progression-free survival (PFS) of all the study patients (N=40). The overall cumulative PFS rate of the study was 71.7% and the overall median progression survival time was 87.14 (range 64.91–109.37) months. (C) Comparison of the cumulative RFS of the patients treated with chemoimmunotherapy (n=24) or rituximab only (n=7). The cumulative RFS of the patients treated with chemoimmunotherapy was 60% and the median relapse-free survival time was observed to be 36.60 months, whereas the cumulative RFS of the patients treated with rituximab only was 50% and the median relapse-free survival time was observed to be 9.67 months. No statistically significant difference was observed between the 2 survival curves (*P*=.29). At 15 months, RFS was 80% for the chemoimmunotherapy group versus 50% for the rituximab group.

The cumulative overall survival of the study participants was 73.4%. The estimated mean overall survival time was 88.29 months (range 66.28-110.31 months). The cumulative PFS of the study sample was 71.7%, with a median PFS time of 87.14 months (range 64.19-109.37; Figure 3).

Survival curves were developed (overall survival and PFS) for all study participants (Figures 3A and B), and a comparative overall survival and PFS KM curve based on various SUVmax values was developed to find the best SUVmax cutoff values that might guide the estimation of survival.

**Figure 4:**
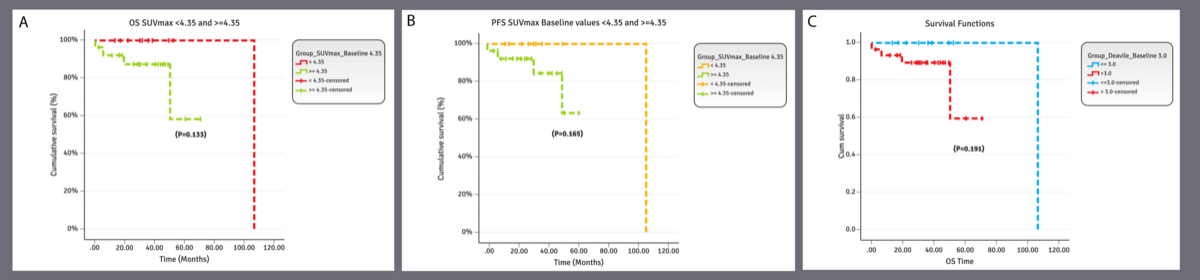
(A) Overall survival maximum SUVmax baseline values <4.35 and ≥4.35. The overall cumulative survival rate of the patients with baseline SUVmax <4.35 was 100% and the median survival time was observed to be 106.67 months, whereas the cumulative survival of the patients with baseline SUVmax ≥4.35 was 58.4% and the median survival time cannot be calculated because the censorship is less than 50%. Moreover, there were no statistically significant differences between the 2 groups (*P*=.13). (B) PFS SUVmax baseline values <4.35 and ≥4.35. The cumulative PFS of the patients with SUVmax baseline <4.35 was 100% and the median survival time was 106.67 months, whereas the cumulative survival of the patients with SUVmax baseline ≥4.35 was 63% and no median survival time was observed. Furthermore, there was no statistically significant difference between the 2 groups (*P*=.17). (C) Overall survival Deauville baseline values ≤3.0 and >3.0. The overall cumulative survival of the patients with baseline Deauville <3.0 was 100% and the median survival time (months) was 106.67, whereas the overall survival of the patients with baseline Deauville ≥3.0 was 59.30% and the median survival time cannot be calculated because the censorship is less than 50%. Moreover, there were no statistically significant differences (*P*=.19) between the 2 groups. OS: overall survival; PFS: progression-free survival; SUVmax: maximum standardized uptake value.

These included survival curves for the baseline PET/CT scan SUVmax ≥4.35 vs <4.35, and ≥9.41 vs <9.41 (33rd and 66th percentile-based values). Using the receiver operating characteristic curve, we were unable to detect an optimum threshold for SUVmax at baseline to predict overall survival; however, we adjudicated that SUVmax value of 4.35 (33rd percentile-based values) was a good cutoff value for segregating survival function at baseline (Figure 4).

**Figure 5:**
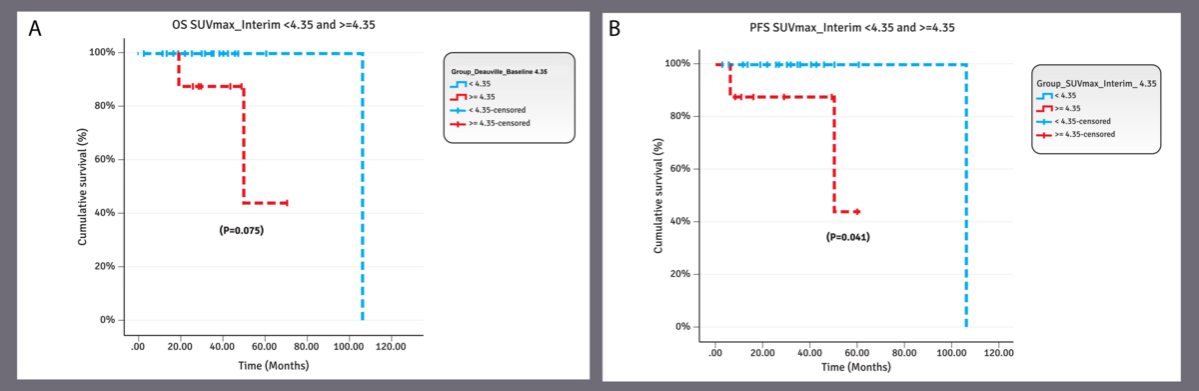
(A) OS SUVmax interim values <4.35 and ≥4.35. The overall cumulative survival of the patients with SUVmax interim <4.35 was 100% and the median survival time was observed to be 106.67 months, whereas the cumulative survival of the patients with SUVmax interim ≥4.35 was 43.8% and the median survival time was 50.37 months. No statistically significant difference was observed between the 2 survival curves (*P*=.08). (B) PFS SUVmax interim values <4.35 and ≥4.35. The cumulative PFS of the patients with SUVmax interim<4.35 was 100% and the median survival time was observed to be 106.67 months, whereas the cumulative survival of the patients with SUVmax interim ≥4.35 was 43.8% and the median survival time was observed to be 50.170 months. Furthermore, statistically significant differences were observed between the 2 survival curves (*P*=.04). OS: overall survival; PFS: progression-free survival; SUVmax: maximum standardized uptake value.

For follow-up phases (after treatment per 6-month follow-up and at 1-year follow-up), we compared overall survival with PFS for SUVmax ≥4.35 vs <4.35 (Figures 5A and B). Comparative overall survival and PFS KM curves for Deauville score ≤3 vs <3 were also developed at presentation (Figure 4C), after treatment per 6-month follow-up, and at 1-year follow-up (not shown).

### The Best Discriminatory SUVmax Values of This Study

Overall, the value of SUVmax (<4.35 vs ≥4.35) on interim PET/CT performed the best discriminatory function in predicting PFS. The cumulative PFS of the patients with SUVmax <4.35 at interim PET/CT was 100% and the observed median survival time was 106.67 months. Whereas the cumulative PFS of the patients with SUVmax ≥4.35 on interim PET/CT was 43.8% and the observed median survival time was 50.17 months. Furthermore, a statistically significant difference was observed between the 2 survival curves (*P*=.04; Figure 5B).

The same value of SUVmax (<4.35 vs ≥4.35) showed good discrimination in predicting overall survival at baseline and interim PET/CT. The cumulative overall survival of the patients with baseline SUVmax <4.35 was 100% and the median survival time was 106.67 months; whereas the cumulative overall survival of the patients with baseline SUVmax ≥4.35 was 58.4% (*P*=.13; Figure 4A). The cumulative overall survival of the patients with an interim SUVmax <4.35 was 100% and the median survival time was observed to be 106.67 months. Whereas the cumulative overall survival of the patients with interim SUVmax ≥4.35 was 43.8% and the median survival time was 50.37 months. Although there was no statistically significant difference between the 2 groups (*P*=.08), there was a trend of better overall survival in the group with interim SUVmax <4.35 (Figure 5A).

The cumulative PFS of the patients with SUVmax baseline<4.35 was 100% and the median survival time was 106.67 months, whereas the cumulative PFS of the patients with baseline SUVmax ≥4.35 was 63%. This difference was not statistically significant *(P*=.17; Figure 4B).

Indolent lymphomas, due to their nature, are deemed to relapse; thus, we did not focus on a detailed analysis of RFS. However, we compared the cumulative RFS of the patients treated with chemoimmunotherapy (n=24) and those of patients treated with rituximab only (n=7). The cumulative RFS of the chemoimmunotherapy group was 60% and the median RFS time was 36.6 months, whereas RFS of the *rituximab-only* group was 50% with a median RFS time of 9.67 months (*P*=.29; Figure 3C).

**Figure 6:**
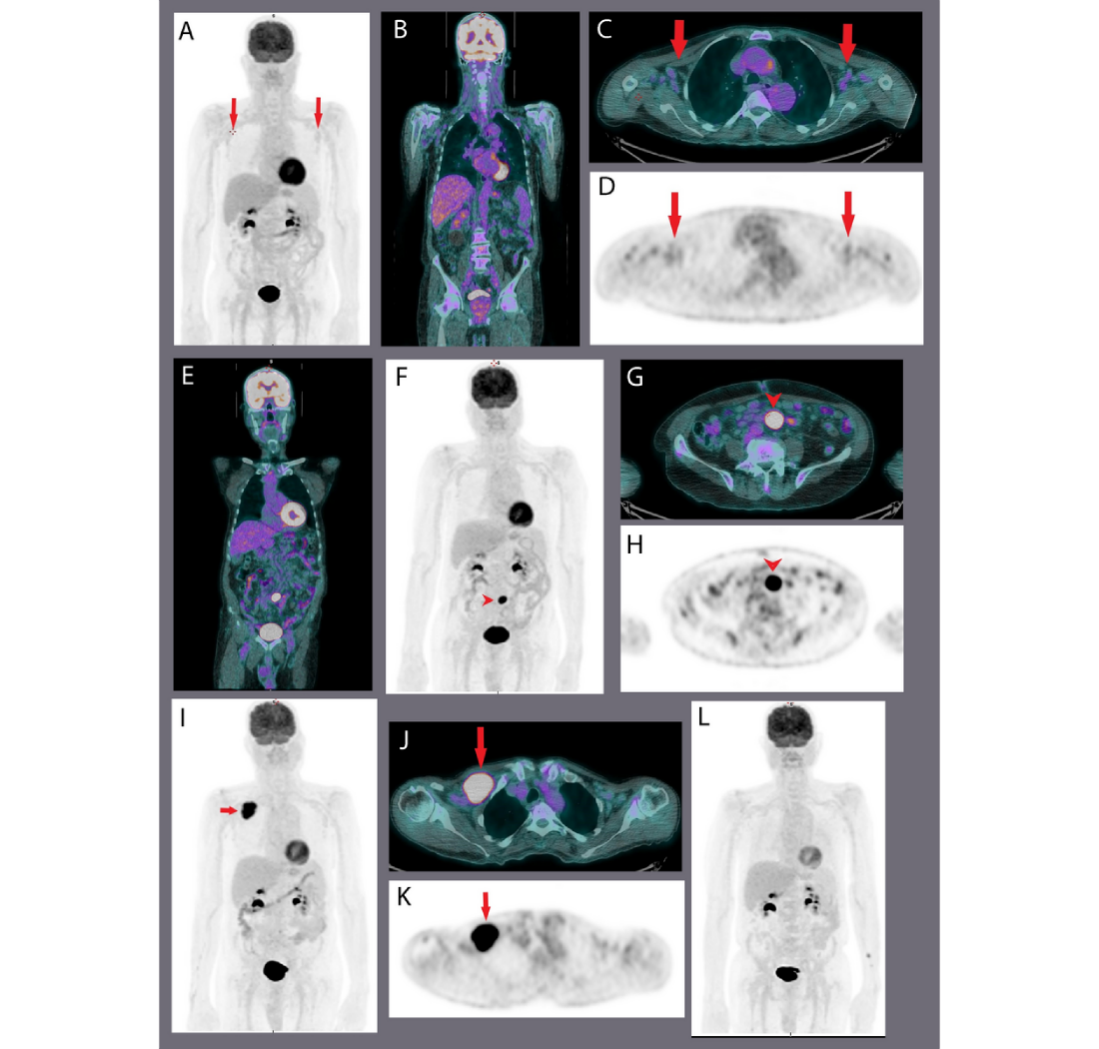
Sequential fluorodeoxyglucose–positron emission tomography/computed tomography (FDG-PET/CT) scan findings in participant number 14, a case of small lymphocytic lymphoma (SLL) and chronic lymphocytic leukemia (CLL) that transformed to diffuse large B-cell lymphoma. Initial presentation showed multiple low FDG-avid lymphadenopathy in the head and neck, mesenteric, bilateral axillary, and bilateral inguinal regions (images A-D). Solid arrows: bilateral axillary lymphadenopathy (A, maximum intensity projection [MIP]; B, coronal fused PET/CT; C, axial fused PET/CT; and D, axial PET). On follow up, interval development of new intense FDG-avid mesenteric lymph nodes (arrowhead in images E-H) and biopsy showed persistent SLL with Ki67 of 10% (E, coronal fused PET/CT; F, MIP; G, axial fused PET/CT; and H, axial PET). The patient sought a second opinion and was lost to follow-up until he reported with interval development of new intense FDG-avid large right subpectoral lymph node and interval resolution of the FDG-avid mesenteric lymph node (images I-K). Solid arrows: right subpectoral lymphadenopathy (I, MIP; J, axial fused PET/CT; and K, axial PET/CT). After R-mini-CHOP (cychlophosphamide, hydroxydaunorubicin, vincristine, and prednisone) chemotherapy, there was complete metabolic response of the previously noted intense FDG-avid subpectoral lymph nodes as well as amild FDG activity in the bilateral axillary lymph nodes (L, MIP).

**Figure 7:**
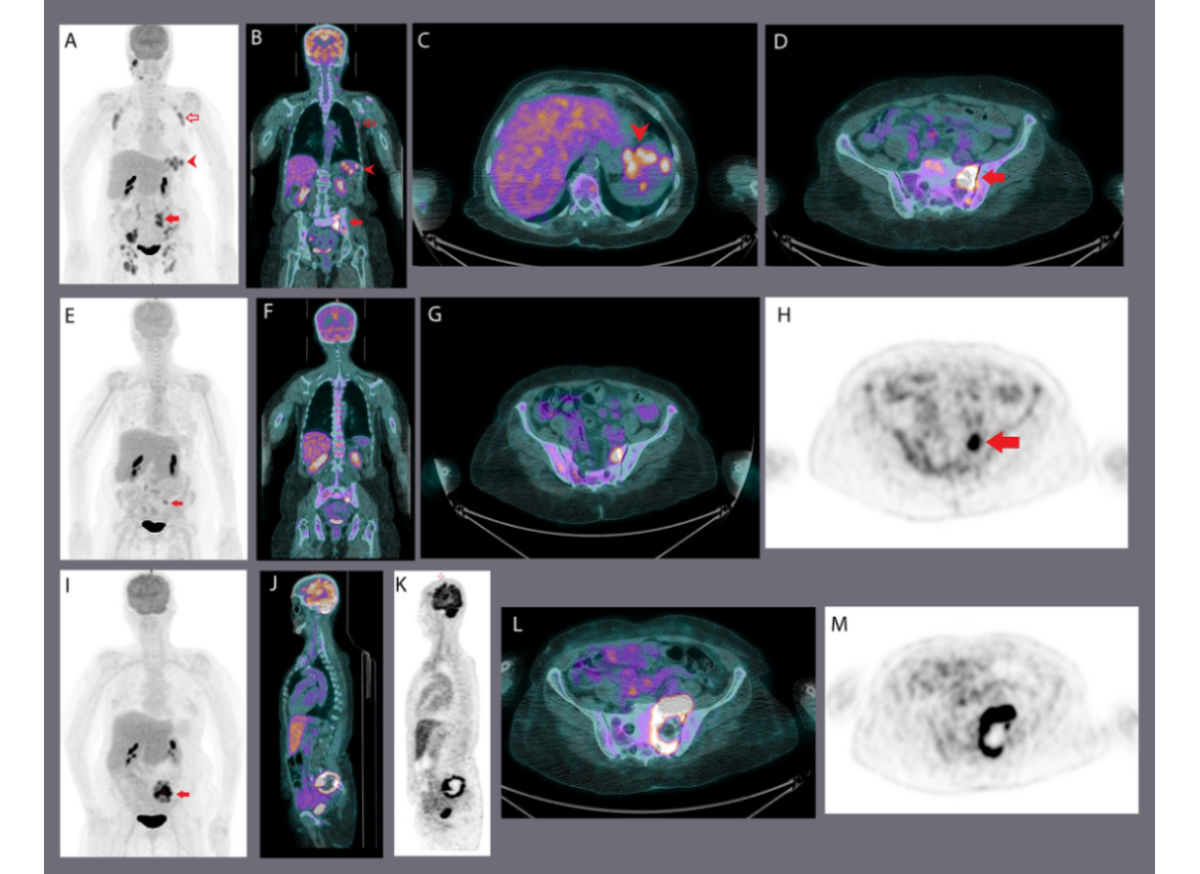
Sequential fluorodeoxyglucose–positron emission tomography/computed tomography (FDG-PET/CT) scan findings in participant number 40, a case of follicular lymphoma that transformed to diffuse large B-cell lymphoma (DLBCL). At baseline, follicular lymphoma causing multiple FDG-avid lymphadenopathy was observed above and below the diaphragm with splenic and osseous involvement (images A-D). Solid arrow: FDG-avid left sacral ala bone metastasis; hollow arrow: FDG-avid left axillary lymphadenopathy; arrowhead: multiple focal areas of FDG uptake in the spleen (A, maximum intensity projection [MIP]; B, coronal fused PET/CT; C, axial fused PET/CT; and D, axial fused PET/CT). After 6 cycles of rituximab + bendamustine, interval metabolic resolution (images E-H) of the previously noted FDG-avid lymphadenopathy and splenic lesion and most of the bony lesions except residual FDG-avid solitary bone lesion was noted in the sacrum (arrows: E, MIP; F, coronal fused PET/CT; G, axial fused PET/CT; and H, axial PET). On further follow-up, an interval increase in size and FDG activity of the left sacral ala metastasis (arrow) was proven to be transformation to DLBCL (images I-M) (I, MIP; J, sagittal fused PET/CT; K, sagittal PET; L, axial fused PET/CT; M, axial PET).

In our study, 3 patients underwent transformation to DLBCL during the study period (eg, Figures 6 and 7). The SUVmax at transformation was 11, 14.3, and 19.8, respectively. This was helpful in guiding targeted biopsies in patients with clinically suspected transformation.

## Discussion

### Principal Findings

In this prospective study evaluating the utility of PET/CT in indolent lymphomas, FDG uptake was measured using the SUVmax and Deauville criteria at various time points. We developed the overall survival and PFS curves for the study population (n=40) and comparative overall survival and PFS KM curves based on SUVmax and Deauville score at baseline and various follow-up phases. Moreover, we evaluated the role of FDG-PET/CT in adaptive therapies, that is, intensive chemotherapy and/or targeted therapy, as well as in situations such as transformation or progression of indolent lymphomas to DLBCL. PET/CT was helpful in detecting SLL and CLL transformation to DLBCL (Figure 6), and FL relapse or transformation to DLBCL (Figure 7).

Median RFS for the rituximab-only group was 50% compared with 60% for the chemoimmunotherapy group, and the median RFS time was 9.67 months and 36.6 months respectively (*P*=.29), indicating insignificant superiority of chemoimmunotherapy. The outcome in patients with FL has improved over the last 2 decades through the introduction of anti-CD20 monoclonal antibodies, which are usually used in combination with chemotherapy. Chemotherapy-free rituximab only has been proposed as a preferable approach in low tumor burden FL, but is still a matter of debate [20-22]. Lockmer et al [23] indicated that an initial rituximab-only approach in patients with indolent lymphomas was associated with an increased overall survival compared with that found in other studies with first-line immunochemotherapy. However, our study, although having a short follow-up time, showed better outcomes with chemoimmunotherapy than with rituximab only.

The most significant findings included an SUVmax cutoff value of 4.35 at interim assessment. The cumulative PFS of the patients with SUVmax interim<4.35 was 100%, and the observed median survival time was 106.67 months as opposed to the patients with SUVmax interim≥4.35 who had a PFS of 43.8% and a median survival time of 50.17 months (*P*=.04). Patients with interim SUVmax <4.35 had an overall survival of 100% with a median survival time of 106.67 months. For patients with interim SUVmax ≥4.35, overall survival was 43.8% with a median survival time of 50.37 months (*P*=.08). Regarding the utility of the Deauville score in indolent lymphomas, we noted a mild trend: at baseline, the overall survival was observed to be better with Deauville score<3.0 compared with Deauville score≥3.0. The cumulative overall survival of the patients with baseline Deauville<3.0 was 100%, whereas the overall survival of the patients with baseline Deauville≥3.0 was 59.30% (*P*=.19). In addition, it confirmed the utility of a remarkably high SUVmax in PET/CT for the transformation of indolent lymphomas in 3 patients.

It can be speculated that a relatively short follow-up period of a small population size divided among 3 management strategies contributed to the marginal degree of segregation of survival curves based on the chosen cutoff values for SUVmax we studied. The median follow-up time of our study population from diagnosis was 33.50 months (mean 34.83 months). The follow-up duration for chemoimmunotherapy patients was 33.24 months (SD 18.2), for the rituximab group it was 35.99 months (SD 11.65), and for the observed group it was 38.83 months (SD 30.82). As indolent lymphomas have a characteristic behavior of being incurable with commonly available therapies, and relapse of the same type of lymphoma or histological transformation to more aggressive types of lymphomas is common, this follow-up period is not sufficient to draw more reasonable results. Expanding the follow-up, as well as increasing the sample size would have yielded statistically robust values.

FDG may be used as a biomarker for assessment of response to therapy by either visual interpretation, such as the DC or by semiquantitative assessment of response using an SUVmax threshold. Currently, ^18^FDG-PET/CT is considered the standard imaging technique for evaluating response assessment in HL, DLBCL, and FL due to its ability to reveal residual metabolic activity irrespective of residual volume. The role of baseline SUVmax in predicting therapeutic outcomes in indolent lymphoma has also been investigated [8]. In our study, SUVmax in FL was 2.1-24 (mean 8). For patients with FL with a high FLIPI score, the SUVmax was 6.1-24 (mean 10.4). All patients with FL, except 1, were alive at the time of the analysis. PFS in FL cases was 90-576 days (mean 780.2 days). For those with high FLIPI, the PFS was 120-1823 (mean 787.3). The overall survival time in all FL cases was 90-1823 (mean, 944.8). In those with a high FLIPI score, the overall survival time was 355-1823 (mean 941.8). Hence, we did not observe the impact of SUVmax on the survival outcome of patients with FL. In concordance with the findings of Trotman et al [17,24], our study, although limited by the number of FL cases (17, of which 5 had bulky disease and 12 had high FLIPI score), we did not observe any correlation between FLIPI score and outcome.

A retrospective study of 81 patients with MCL revealed that baseline SUVmax is predictive of outcome; in patients with SUVmax <5.0, the 5-year survival was 87.7%, whereas it dropped to 34% when SUVmax was>5.0 [25]. In our study, 3 patients had MCL, and their baseline SUVmax ranged from 5.2-6.8. The overall survival time ranged from 181 to 1379 days (median 1051). Although all 3 had advanced-stage disease and received similar treatment with rituximab and bendamustine, one patient with a baseline SUVmax of 5.2 died without achieving complete metabolic remission, reflecting no meaningful correlation between SUVmax and survival in this limited number of patients.

Currently, there is no consensus on the optimal discriminatory baseline SUVmax for indolent lymphomas. Schoder et al [26] found that all patients with indolent non-HL had an SUVmax ≤13 and that SUVmax>10 was the best cutoff value to discriminate between aggressive and indolent lymphoma with 81% specificity. In our study, we included only confirmed indolent lymphoma cases, and we observed a median SUVmax of 6.35 (2.1 to 15.4) at presentation.

In a prospective study of 38 patients with clinical or histological suspicion of transformed lymphoma, a biopsy of the site with the highest SUVmax showed that all patients with SUVmax>17 had transformed, whereas those with SUVmax <17 had not. The best cutoff value was SUVmax of 14 [27]. In our study, 3 patients underwent transformation during the study period. The SUVmax at transformation ranged from 11 to 19.8 (mean=13.7), which led to a decision of guided biopsy. Although our study size is small, the findings in our study corroborate observations by Barrington et al [28], that FDG-PET/CT can guide targeted biopsies in patients with suspected transformation.

In 2014, the imaging group of the International Conference on Malignant Lymphoma recommended that evaluation of response to treatment of FDG-avid lymphoma should be performed with FDG-PET/CT [28], and FL is considered a lymphoma subtype for which FDG-PET/CT is indicated as mandatory for staging and response assessment. The DC proposed in 2009 are now commonly accepted for PET/CT reporting for response assessment. An advantage of the DC is that a graded response is defined that allows the threshold to be changed according to the proposed intervention: escalation (PET/CT-positive if score ≥4) or de-escalation (PET/CT-positive if score ≤3) to select true-positive or true-negative patients. It has been proposed that the DC described initially for reporting interim PET/CT may also be used for reporting end-treatment PET/CT [28,29].

The FDG-PET/CT reporting using DC has been used to tailor therapy at the end of treatment and during treatment with a PET/CT-guided strategy in HL and other lymphomas including FL [10,30]. The International Conference on Malignant Lymphoma imaging committee proposed that the DC be extended to end-treatment evaluation [28,31]. With rapid improvements in scanner technology, the minimum score requirement for PET/CT used in the International Harmonization Project (IHP) has become less relevant, and the mediastinal threshold used in the IHP is analogous to a DC score of 1 or 2. Furthermore, a single method of visual assessment in PET/CT is desirable for the interim and end-treatment response assessments. DC scores 1-3 are therefore considered to represent a complete metabolic response under standard treatment. The validity of using the DC with this cutoff for end-treatment PET/CT reporting has recently been confirmed in important studies in FL with a high tumor burden [17].

Federico et al [10] compared the diagnostic accuracy of FDG-PET/CT for clinical evaluation at the end of treatment using the DC and IHP criteria]; their results indicated that the DC is simpler to apply and certainly more effective than the IHP criteria [10].

The results of all the studies discussed above suggest that this holistic approach could improve risk stratification in FL and other indolent lymphomas, and may help build new treatment strategies. However, larger series and prospective multicenter studies are needed before they can be used as risk factors. PET/CT has recently been reported to be useful in detecting BM involvement in FL [32]. However, in our study, of the 17 patients with FL, 12 (70.6%) had BM involvement in the biopsy report, but we could not detect BM involvement in one of those patients, as depicted in Figure 6.

Although using PFS versus overall survival and other quality measures [33] is a matter of debate, we estimated both overall survival and PFS in this prospective study. It seems clear that the growing use of PFS as a primary endpoint in many randomized controlled trials of advanced solid tumors is because a definition for progression exists, we can measure it, and it shortens trial periods. We also used a more meaningful overall survival, but obviously, it requires longer follow-up for a better picture.

The PRIMA trial [34] showed a positive PET/CT scan after treatment, predicted a shorter PFS, and a higher risk of death [24]. A subsequent prospective observational study with a standardized PET/CT acquisition and interpretation criteria confirmed these findings, with a similar risk of progression for PET/CT-positive patients who were assessed centrally by applying a cutoff of ≥4 on DC [35].

To compare the central PET/CT review with that of the local investigators for a subset of patients with FL in the PRIMA trial using 2 standardized response criteria, the 2007 IHP criteria and the DC, Tychyj-Pinel, et al [9] reported that at diagnosis, the mean SUVmax of PET-positive cases was 11.7 (range 4.6–35.6), as compared with a mean SUVmax of 8 (2.1-24) in our FL cases. In both studies, there was no significant association between baseline SUVmax and PFS. In 60 postimmunochemotherapy induction scans, Tychyj-Pinel, et al [9], applying the DC with a cutoff ≥4, reported a significantly inferior 42-month PFS in PET-positive patients of 25% versus 61.4% in PET-negative patients (*P*=.01). In our study, only 15 patients with FL underwent postinduction PET/CT scans; 2 patients had a DC score of 5 and their mean PFS was 760 days, whereas 13 patients with a DC score ≤3 had a mean PFS of 782.35 days. We observed no significant difference in PFS across these DC scores (*P*=.39). Although in our study population, we could show some trends in overall survival and PFS in relation to SUVmax and DC, we could not obtain the optimal pretreatment SUVmax cutoff for receiver operating characteristic analysis.

### Ideas for Future Research

What else can be done from such a study-related data in the future in view of ongoing alternative efforts in the field–food for thought? As we planned our study and started it, multiple developments in the alternate analysis of PET/CT images have taken place. Although these developments are attractive, due to logistic difficulties, this analysis could not be performed. Some of these ideas are described below in future studies.

Texture analysis and data mining of medical images, also known as *Radiomics,* is an emerging field in radiology. Radiomics is the extraction of occult information from clinical images and subsequent data mining to gain actionable insights. The radiologic texture is the variation in image intensities and occult patterns within an image and is an important part of Radiomics [36,37].

Other exciting developments include integrative FDG-PET/CT, combining a PET/CT scan performed at different time points during treatment or quantitative PET/CT scan parameters with clinical or biological data or other imaging techniques. The integration of information from several different sources (clinical, biological, imaging, etc, along with or integrating with ΔSUVmax [change in SUVmax between 2 points] could lead to more personalized risk stratification) [29].

The prognostic value of total metabolic tumor volume (TMTV) has also been investigated. Trotman et al [17] observed that in patients with FL with a high tumor burden, a TMTV>938 cm^3^ can identify a small subset of patients with a poor outcome [17].

Total lesion glycolysis, which is the sum of the product of the metabolic volume of each local tumor multiplied by its mean standardized uptake value (total lesion glycolysis = Σmetabolic tumor volume × mean standardized uptake value), is another parameter that has been studied in DLBCL as a better prognostic tool compared with TMTV [13,14]. It may be attractive to study TMTV in indolent lymphomas.

Finally, keeping the above advancements in mind, our study calls for action in our region for future prospective studies.

### Limitations

The limitations of our study include the small sample size divided among the 3 management strategies and a limited period of follow-up in these indolent lymphomas. Making any firm conclusions about the utility of PET/CT based on a small study population and heterogeneous patients is difficult.

### Conclusions

In this prospective cohort study consisting of 40 patients, we demonstrated the utility of PET/CT in indolent lymphomas. Overall, the values of SUVmax (<4.35 vs ≥4.35) on interim PET/CT performed the best discriminatory function in predicting PFS. The cumulative PFS of the patients with SUVmax <4.35 at interim PET/CT, was 100% and the median survival time was 106.67 months, whereas the cumulative PFS of patients with SUVmax ≥4.35 was 43.8% and the median survival time was 50.17 months (*P*=.04). The same values showed a good trend in predicting overall survival at baseline and interim PET. Further prospective studies in this region is needed.

## Abbreviations

BM: bone marrow
CLL: chronic lymphocytic leukemia
CT: computed tomography
DC: Deauville criteria
DLBCL: diffuse large B-cell lymphoma
FDG: fluorodeoxyglucose
FL: follicular lymphoma
FLIPI: follicular lymphoma international prognostic index
HL: Hodgkin lymphoma
IHP: International Harmonization Project
KM: Kaplan–Meier
MCL: mantle cell lymphoma
MIP: maximum intensity projection
PET: positron emission tomography
PFS: progression-free survival
R-CHOP: rituximab-cychlophosphamide, hydroxydaunorubicin, vincristine and prednisone
R-CVP: cituximab-cychlophosphamide, vincristine and prednisone
RFS: relapse-free survival
SLL: small lymphocytic lymphoma
SUVmax: maximum standardized uptake value
TMTV: total metabolic tumor volume
